# The Impact of Protein Feed on the Urea-to-Creatinine Ratio—A Retrospective Single-Center Study

**DOI:** 10.3390/nu17081293

**Published:** 2025-04-08

**Authors:** Petra Hillinger, Andreas Markl-Le Levé, Simon Woyke, Marco Ronzani, Janett Kreutziger, Stefan Schmid, Christopher Rugg

**Affiliations:** Department of Anaesthesiology and Intensive Care Medicine, Medical University of Innsbruck, Anichstrasse 35, 6020 Innsbruck, Austria; petra.hillinger@tirol-kliniken.at (P.H.); christopher.rugg@tirol-kliniken.at (C.R.)

**Keywords:** urea-to-creatinine-ratio, protein metabolism, catabolism

## Abstract

Background/Objectives: The urea-to-creatinine ratio (UCR) serves as a biochemical marker for catabolism in the intensive care unit (ICU). UCR increases mainly due to an elevated urea generation from increased protein metabolism. This study aimed to evaluate the impact of protein intake on UCR progression in ICU patients. Methods: The inclusion criteria of this retrospective, single-center analysis required an ICU stay of at least 14 days without requirement of renal replacement therapy (n = 346 patients). Patients were grouped based on daily mean protein intake per kilogram between days 5 and 14: low (≤0.8 g/kg/d, n = 120), medium (0.9–1.2 g/kg/d, n = 132), and high (≥1.3 g/kg/d, n = 94). Data on daily protein and calorie intake, calorie deficit, urea generation rate, serum creatinine, urea, UCR and creatinine clearances were analysed. Risk factors for developing a high UCR were determined via logistic regression analysis adjusted for sex, age, bodyweight, disease severity (SAPS III admission score) as well as mean protein intake and calorie deficit during day 5 and 14 on ICU. Results: Higher protein intake was associated with increased calorie intake, lower calorie deficit, and led to an elevated urea generation rate and higher UCR. Renal function and serum urea trends were comparable between all groups, while creatinine was significantly lower in the high-protein group. Risk factors for developing an elevated UCR included older age, female sex and higher protein intake. Conclusions: An elevated UCR in the early ICU phase may indicate an increased protein metabolism, not only deriving from catabolism but also from a high protein feed.

## 1. Introduction

Muscle wasting is a common and serious complication in patients suffering from persistent critical illness [1,2]. Multiple organ dysfunction syndrome (MODS) triggers a profound catabolic response, which can lead to significant and rapid muscle mass loss. Reductions of up to 1 kg per day within the first 10 days of ICU stay have been reported in patients with MODS [3,4]. This rapid muscle degradation is a hallmark of critical illness and contributes significantly to poor patient outcomes, including prolonged recovery times, decreased physical function, and increased mortality [5,6,7].

Muscle catabolism leads to an elevated urea generation, while at the same time the increasing decline in muscle mass leads to a decrease in creatinine generation, hence leading to an increase in the urea-to-creatinine ratio [8]. An elevated urea-to-creatinine ratio (UCR) has been shown as a potential biochemical indicator of muscle catabolism [9]. Additional factors contributing to increased urea production include dietary protein intake and gastro-intestinal bleeding [10]. In patients with persistent critical illness, urea levels can additionally be elevated due to factors such as increased urea reabsorption induced by hypovolemia and decreased urea excretion resulting from acute kidney injury [8,9].

Given the detrimental impact of muscle wasting on recovery, addressing the underlying catabolic processes remains a key challenge in the management of critically ill patients, underscoring the importance of early recognition and targeted interventions [11,12].

High-protein delivery, combined with active early rehabilitation, supports muscle volume maintenance in critically ill patients [13]. A high protein intake might help reduce muscle mass breakdown in critically ill patients [14,15] and could potentially lower the UCR by decreasing the amount of urea generated from the breakdown of endogenous proteins. This would suggest that providing sufficient protein supplementation could support preservation of muscle mass and thus mitigate catabolic processes in ICU patients [13,14,15,16].

On the other hand, a high protein intake could also stimulate increased protein metabolism, leading to a higher urea production from the exogenous protein provided. This increase in urea generation could elevate the UCR, despite the intended benefit of preserving muscle mass. Therefore, while high protein intake may protect against muscle loss, it could paradoxically result in a higher UCR due to the increased burden of protein metabolism [16,17].

The overall impact of high protein intake on UCR may be determined by the dynamic balance between two competing processes: the preservation of muscle mass, which could help lower UCR by reducing endogenous protein breakdown, and the increased metabolic activity associated with processing the additional dietary protein, which may raise urea levels. Understanding how these factors interact is essential for optimizing nutritional strategies in critically ill patients. In our study, we aimed to investigate the impact of protein intake on the progression of UCR in ICU patients. Specifically, we sought to evaluate how varying levels of protein supplementation influence UCR dynamics over time. Understanding this relationship could provide valuable insights into optimizing nutritional strategies for critically ill patients.

## 2. Materials and Methods

This retrospective study was approved by the Ethics Committee of the Medical University of Innsbruck (EK Nr.: 1023/2024) and took place at the traumatological as well as the general and surgical intensive care unit of the Department for Anaesthesiology and Intensive Care Medicine of the Innsbruck Medical University Hospital. Approximately 700–800 mainly surgical (cardiac, visceral, thoracic, transplant, orthopaedics and traumatology) but also medical patients are treated annually on 23 level 3 beds in total.

**Figure 1:**
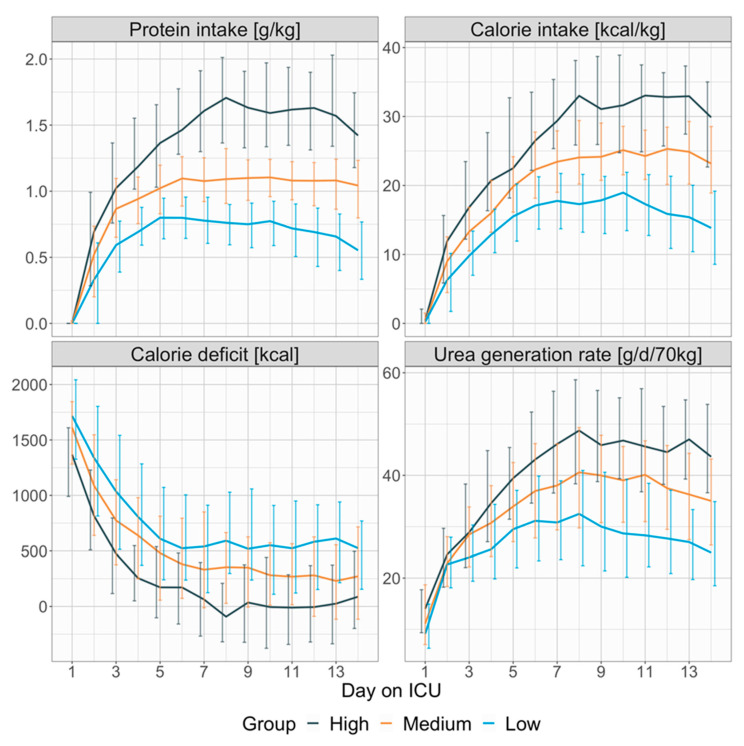
Group dependent presentation of daily protein intake, calorie intake, calorie deficit and urea generation rate. Lines represent daily medians, vertical error bars the interquartile range. Calorie deficit is calculated as difference between energy expenditure (generated from ventilator derived VCO_2_) and total calorie intake. Urea generation rate is calculated as sum of total urinary urea excretion and body urea variation (difference in serum urea and serum urea the day before multiplied with 0.6 times the bodyweight). For better comparison, the urea generation rate was normalized to 70 kg by dividing the urea generation rate with the patient’s bodyweight and multiplying the result with 70 kg.

Patients with digital ICU documentation in our Patient Data Management System (PDMS) within a 11.5-year timeframe from 1 January 2011 to 31 July 2022 were extracted. Digital ICU documentation was implemented on one ICU ward in 2007, while the other ward adopted digital documentation in 2018. The primary data pool comprised 5691 ICU patients. Patients with an ICU stay of less than 14 days (n = 4510) were excluded from the analysis, along with patients requiring renal replacement therapy during their ICU stay (n = 667) and those with missing bodyweight data (n = 168). The remaining 346 patients were categorized into three groups based on their daily average protein intake per kilogram of body weight between ICU days 5 and 14: low intake (≤0.8 g/kg/d, n = 120), medium intake (0.9–1.2 g/kg/d, n = 132), and high intake (≥1.3 g/kg/d, n = 94; Figure 1). The general observation period spanned ICU days 1 to 14 (study flow chart, Supplemental Figure S1).

Patient data were obtained from the hospital information system and the ICU PDMS archive. The collected data included demographic details (age, sex, bodyweight, height), admission characteristics (elective surgery, emergency surgery/admission), and information regarding the treating medical discipline (cardiac surgery, visceral, thoracic and transplant surgery, orthopaedics and traumatology, internal medicine, or other). Additionally, the Simplified Acute Physiology Score III (SAPS III) [18] on ICU admission and the length of ICU stay were recorded. Protein and calorie intake were calculated based on the amounts provided through enteral and parenteral nutrition (Supplemental Figure S2), as well as from non-nutritional sources such as glucose solutions (e.g., maintenance fluids and drug solvents) and propofol [19].

Energy expenditure (EE) was estimated using ventilator-derived VCO_2_ and the modified Weir equation, assuming a respiratory quotient of 0.89 (EE = daily mean VCO_2_ [mL/min] × 7.975) [20]. The calorie deficit was determined by comparing the total calorie intake to the estimated energy expenditure and calculating the difference. The urea generation rate was derived as sum of total urinary urea excretion and body urea variation (urine urea concentration [g/L] × 24 h-urine output [L] + (serum urea_day(i+1)_ [g/L] − serum urea_day(i)_ [g/L]) × 0.6 [L/kg] × bodyweight [kg]). For standardized comparisons, the urea generation rate was normalized to a reference body weight of 70 kg by dividing the calculated rate by the patient’s actual body weight and multiplying the result by 70 kg.

Daily urine analyses are performed routinely from 24-h urine collection from every patient in our department. Therefore, true creatinine clearances were accessible and normalized to 1.73 m^2^ by dividing calculated creatinine clearances with the patient’s actual body surface area (Du Bois method [21]: 0.007184 × (height in cm)^0.725^ × (bodyweight in kg)^0.425^) and multiplying the result with 1.73 m^2^. The urea-to-creatinine ratio was derived using serum concentrations of urea and creatinine (UCR = serum urea [mg/dL]:serum creatinine [mg/dL]).

Statistical analysis was performed using R (v4.3.2, R Core Team) and RStudio (v2023.12.0-369, RStudio, Inc., Boston, MA, USA). Due to non-normal distribution (Shapiro-Wilk test), data is presented as count and percentage or median and interquartile range (Q1–Q3), as appropriate. Differences in categorical variables between groups were analysed using the Chi-squared test, while differences in continuous variables were assessed using the Kruskal-Wallis test. Graphical presentation of daily trends in medians and interquartile ranges or as generalized additive model was conducted utilizing the R package ggplot2. Risk factors for developing an elevated UCR (defined as a mean UCR > 75 mg/dL:mg/dL between ICU days 5 and 14) were evaluated using a logistic regression model. The analysis was adjusted for age, sex, disease severity at admission (SAPS III), bodyweight, and mean calorie deficit and protein intake during the same period (ICU days 5 to 14). Multicollinearity was assessed and excluded by variance inflation factor analysis (R package: car) and model fit was evaluated and confirmed via McFadden’s pseudo-R^2^. The results are reported as odds ratios (OR) with corresponding 95% confidence interval (CI). A *p*-value < 0.05 was considered statistically significant.

## 3. Results

### 3.1. General Demographics and Group Characteristics

From 5691 eligible patients with digital ICU documentation in the queried timeframe, 346 remained after exertion of inclusion and exclusion criteria. Regarding group allocation, 120 patients were assigned to the low group (mean protein intake between ICU-day 5 and 14: ≤0.8 g/kg/d), 132 patients to the medium group (0.9–1.2 g/kg/d) and 94 to the high group (≥1.3 g/kg/d). Significantly less female patients were present in the low and medium group when compared to the high group (n = 24 (20.0%) vs. n = 38 (28.8%) vs. n = 43 (45.7%); *p* < 0.001; Table 1). Accordingly, bodyweight (88.9 (78.8, 100.0) kg vs. 76.0 (68.4, 84.0) kg vs. 63.1 (52.6, 75.0) kg; *p* < 0.001) and height differed significantly as well. There were no significant differences between the protein intake groups regarding age, admission type, or medical discipline. However, patients in the low protein intake group had a lower severity of illness, as indicated by their SAPS III, compared to the medium and high intake groups (58.0 (51.0, 70.0) vs. 61.0 (54.8, 71.0) vs. 67.0 (62.0, 77.0); *p* < 0.001, Table 1). Similarly, the length of stay in the ICU was shorter for the low group than for the medium and high groups (19.0 (15.0, 24.3) vs. 21.0 (17.0, 31.0) vs. 21.0 (18.0, 27.0); *p* < 0.001).

### 3.2. Protein- and Calorie-Intake

Regarding daily protein- and calorie-intake as well as daily calorie deficit and urea generation rate, numerical values are presented in Supplemental Table S1 and graphical depiction is shown in Figure 1. Protein- and calorie-intake differed significantly between groups from day 2 onwards (e.g., day 8: 0.76 (0.59, 0.9) g/kg vs. 1.09 (0.91, 1.32) g/kg vs. 1.71 (1.36, 2.01) g/kg; *p* < 0.001 and 17.29 (13.22, 21.61) kcal/kg vs. 24.06 (20.2, 29.42) kcal/kg vs. 33 (25.87, 38.12) kcal/kg; *p* < 0.001). Significant group differences were present from day 1 onwards regarding calorie deficit (e.g., day 8: 591.46 (296.67, 1029.00) kcal vs. 353.37 (28.04, 664.93) kcal vs. −92.38 (−319.26, 208.36) kcal; *p* < 0.001) and day 3 regarding urea generation rate (e.g., day 8: 32.49 (22.4, 40.96) g/d/70 kg vs. 40.59 (29.75, 49.32) g/d/70 kg vs. 48.74 (38.34, 58.64) g/d/70 kg; *p* < 0.001). Regarding calorie balance, the high protein intake group showed a shift towards a calorie surplus, with median values turning negative on days 8, 10, 11, and 12.

Patients in the low protein intake group had higher insulin requirements with comparable phosphate requirements between all groups (Supplemental Figure S2).

### 3.3. Renal Function and the Urea-to-Creatinine Ratio

**Figure 2:**
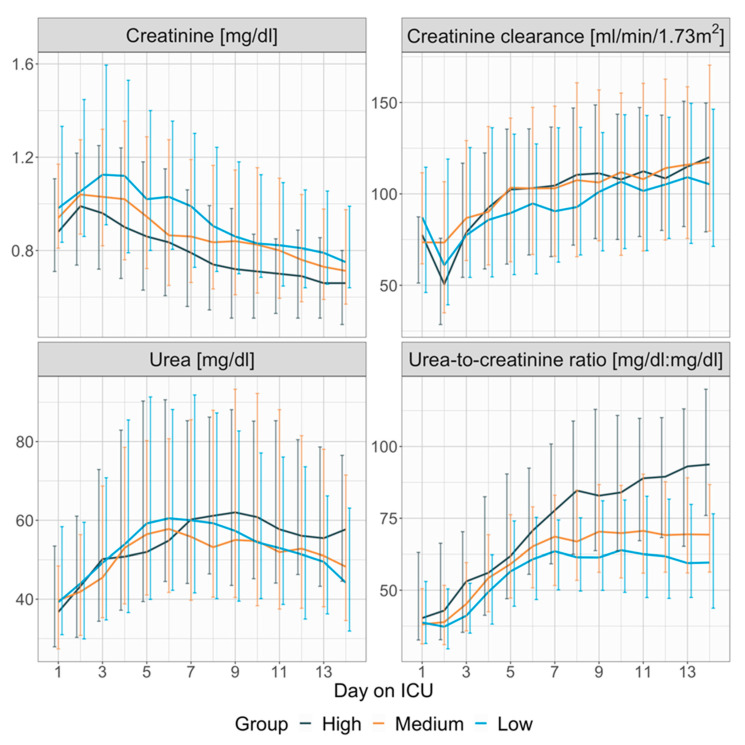
Group dependent presentation of serum creatinine, creatinine clearance, serum urea and serum urea-to-creatinine ratio. Lines represent daily medians, vertical error bars the interquartile range. Creatinine clearance was measured by full 24-h urine collection and then normalized to 1.73 m^2^ body surface area by dividing the measured creatinine clearance with the patient’s actual body surface area and multiplying the result with 1.73 m^2^.

Daily values for serum creatinine, urea, urea-to-creatinine ratio, and creatinine clearance are provided in detail in Supplemental Table S2 and illustrated in Figure 2. Serum urea levels and creatinine clearance measurements were largely consistent across the groups throughout the observation period. Serum creatinine levels were consistently lower in the high protein intake group from days 1 to 14, while the urea-to-creatinine ratio was significantly higher in this group during days 2 to 4 and 6 to 14 (Figure 2). For example, on day 8, serum creatinine levels were 0.9 (0.71, 1.24) mg/dL in the low protein intake group vs. 0.84 (0.64, 1.16) mg/dL in the medium group vs. 0.74 (0.54, 0.99) in the high protein intake group (*p* = 0.001, Supplemental Table S2).

UCR was 61.45 (49.7, 75.16) in the low protein intake group vs. 66.87 (53.44, 84.66) in the medium group vs. 84.65 (62.46, 108.87) in the high protein intake group (*p* < 0.001, Supplemental Table S2).

### 3.4. Risk Factors for Developing a High Urea-to-Creatinine Ratio

A high urea-to-creatinine ratio was defined as a mean UCR between day 5 and 14 above 75. Multivariate logistic regression analysis adjusted for age, sex, bodyweight, SAPS III as well as mean calorie deficit and protein intake between day 5 and 14 on ICU, revealed age (OR 1.05 (1.03, 1.07) per year increase; *p* < 0.001), female sex (male vs. female OR 0.45 (0.25, 0.83); *p* = 0.010) and protein intake (1.16 (1.05, 1.28) per 0.1 g/kg increase; *p* = 0.003) to be significant risk factors for developing a high urea-to-creatinine ratio (Table 2). Model fit quality was confirmed with a McFadden’s pseudo R^2^ of 0.30. Negative effects through multicollinearity were ruled out by variance inflation factor analysis (highest VIF 1.71).

## 4. Discussion

In this study, a higher protein intake was associated with an increased calorie intake and led to an elevated urea generation rate and higher UCR. While renal function was comparable between groups, risk factors for developing an elevated UCR included older age, female sex and higher protein intake.

Our findings underscore the complex relationship between protein intake, metabolism, and catabolic activity in critically ill patients. While protein supplementation is generally intended to counteract muscle breakdown and support recovery, its effectiveness remains controversial. In reality, adequate protein intake primarily aims to mitigate the negative consequences of malnutrition [17,22,23,24,25]. However, the observed increase in UCR with higher protein intake suggests that this strategy may have unintended consequences. Calorie and protein intake do not necessarily equate to their absorption or effective utilization by the body; therefore, the interpretation of calorie deficits should be considered with caution [22]. The urea generation rate, when multiplied by 2.8, represents the amount of protein metabolized (based on urea weight-wise containing 45% and an average protein mix containing about 16% nitrogen). In this study, the amount of metabolized protein often exceeded the protein intake and continued to increase with higher protein supplementation. This indicates that catabolism persists and protein metabolism is further stimulated by protein administration [11,17]. The rise in UCR with high protein intake highlights a persistent catabolic state, as metabolized protein consistently exceeds intake [8]. This phenomenon may indicate that while protein supplementation stimulates metabolism, it does not fully mitigate catabolism [26]. The concurrent decline in creatinine levels, despite increased protein intake, further supports the presence of an ongoing, potentially irreversible catabolic state. This paradoxical outcome warrants careful consideration, particularly when designing nutrition protocols for ICU patients.

The analysis identifies female sex, higher age, and high protein intake as significant risk factors for an elevated UCR. Importantly, calorie deficits were not associated with increased UCR, suggesting that increased nutritional and protein intake, rather than underfeeding, is more likely to exacerbate catabolism in this context [17,26]. Women in the high-protein group, who typically weigh less and have lower creatinine levels [27], along with elderly patients, who physiologically have declining creatinine levels [28], appear more susceptible to excessive protein and calorie supplementation due to standardized nutrition schemes that do not account for individual energy requirements. This observation raises concerns about the adequacy of one-size-fits-all approaches in ICU nutrition and highlights the need for tailored protocols [29].

The proportion of parenteral nutrition relative to total energy intake was nearly identical across groups during the first week, and even decreased during the second week in the high-protein group—which appeared to have better nutritional tolerance.

Patients in the low-protein group required higher daily insulin doses. The observed difference could—at least partially—be explained by poorer nutritional tolerance in this group. Phosphate requirements were comparable across all groups.

In this study, energy expenditure was estimated using ventilator-derived VCO_2_ and the modified Weir equation. Information on body composition with measurements of muscle and fat masses are not available. Interpreting weight changes independently would be complicated by fluid retention, which may differ between patients. Patients in the high-protein group, whose median body weight is significantly lower than that of the other groups, produce more urea in response to nitrogen intake. Although urea production is increased, their presumably lower muscle mass results in inherently lower creatinine production, potentially distorting the UCR.

The interplay between high protein intake, catabolism, and UCR raises questions about the optimal protein dosing in critically ill patients. The potential drawbacks of increased urea production and heightened UCR must be weighed carefully. Noteworthy, an elevated UCR has not only been shown to be a surrogate parameter for protein metabolism but has also been shown to be associated with the development of hypernatremia in the ICU via increased urea-induced osmodiuresis [30,31,32]. Tailored nutrition protocols that consider individual risk factors, such as sex and body weight, may help mitigate the risks of overfeeding and its metabolic consequences.

A major challenge in interpreting the UCR in critically ill patients remains the numerous factors influencing it, such as kidney function, muscle metabolism, catabolism, and protein intake. Daily protein intake should be considered in further evaluation to avoid misinterpretation.

Limitations of our study include its single-center, retrospective design and the lack of information regarding confounding factors such as hydration status and the amount of exogenous or ingested protein. Accurately and objectively measuring hydration status in a clinical setting is challenging. In our ICU setting, hydration status and fluid requirements are typically assessed using invasive methods (e.g., PiCCO) or non-invasive techniques such as echocardiography, pulse pressure variation (PPV), stroke volume variation (SVV), the passive leg raise test, and central venous pressure (CVP), despite its known limitations. For the purposes of our analysis, we assumed that general treatment protocols, including fluid management strategies, were comparable across patients.

Further research is needed to determine the optimal protein dosing in critically ill patients and to analyse the potential drawbacks of increased urea production and a corresponding rise in UCR. Specifically, studies should explore the long-term outcomes of high protein supplementation and evaluate alternative strategies to balance muscle preservation with metabolic stability like early mobility and resistance exercise [33,34].

To gain a deeper understanding of the complex interactions, future studies could focus on the influence of acute kidney injury and gender on the UCR.

## 5. Conclusions

Highlighting the complex interplay between protein intake and muscle catabolism in critically ill patients, this study shows that an elevated UCR in the early ICU phase may indicate an increased protein metabolism, not only deriving from catabolism but also from a high protein feed. As particularly female and elderly patients seem to be at risk of excessive protein supplementation, our findings underscore the need for individualized protein dosing strategies in the ICU, carefully balancing the benefits of avoiding protein malnutrition against the metabolic impact of protein supplementation. Further research is essential to refine nutritional guidelines and optimize outcomes for critically ill patients, particularly those at heightened risk of overfeeding or metabolic complications.

## Figures and Tables

**Table 1:** Group characteristics.

	Low (n = 120)	Medium (n = 132)	High (n = 94)	*p* Value
Sex				<0.001
Female	24 (20.0%)	38 (28.8%)	43 (45.7%)	
Male	96 (80.0%)	94 (71.2%)	51 (54.3%)	
Age [years]				0.662
Median (Q1, Q3)	56.0 (46.8, 69.0)	57.0 (41.0, 68.3)	59.5 (43.3, 69.0)	
Bodyweight [kg]				<0.001
Median (Q1, Q3)	88.9 (78.8, 100.0)	76.0 (68.4, 84.0)	63.1 (52.6, 75.0)	
Height [cm]				<0.001
Median (Q1, Q3)	175.333 (167.8, 181.0)	173.0 (167.2, 179.0)	170.0 (163.5, 177.0)	
Admission type				0.563
Elective	42 (35.0%)	38 (28.8%)	29 (30.9%)	
Emergency	78 (65.0%)	94 (71.2%)	65 (69.1%)	
Medical discipline				0.730
Cardiac surgery	20 (17.7%)	17 (14.2%)	9 (10.1%)	
Orthopaedics/Traumatology	28 (24.8%)	35 (29.2%)	19 (21.3%)	
Visceral surgery	13 (11.5%)	13 (10.8%)	14 (15.7%)	
Internal medicine	29 (25.7%)	28 (23.3%)	25 (28.1%)	
Other	23 (20.4%)	27 (22.5%)	22 (24.7%)	
Missing	7	12	5	
SAPS III admission score				<0.001
Median (Q1, Q3)	58.0 (51.0, 70.0)	61.0 (54.8, 71.0)	67.0 (62.0, 77.0)	
ICU length of stay [days]				<0.001
Median (Q1, Q3)	19.0 (15.0, 24.3)	21.0 (17.0, 31.0)	21.0 (18.0, 27.0)	

**Table 2:** Logistic regression analysis. Odds ratios for developing a high urea-to-creatinine ratio (mean between day 5 and 14 on ICU > 75).

High vs. Low Urea-to-Creatinine Ratio	Crude OR (95% CI)	Adjusted OR (95% CI)	*p*
Calorie deficit per 100 kcal increase	0.85 (0.80, 0.90)	0.94 (0.88, 1.00)	0.052
Sex male vs. female	0.30 (0.18, 0.50)	0.45 (0.25, 0.83)	0.010
Age per year increase	1.05 (1.03, 1.06)	1.05 (1.03, 1.07)	<0.001
Bodyweight per kg increase	0.97 (0.96, 0.98)	0.99 (0.97, 1.01)	0.375
SAPS III admission score per 1 point increase	1.03 (1.01, 1.05)	1.00 (0.98, 1.02)	0.82
Protein intake per 0.1 g/kg increase	1.20 (1.12, 1.28)	1.16 (1.05, 1.28)	0.003

## Data Availability

The data that support the findings of this study are available from the corresponding author upon reasonable request.

## Supplementary Materials

The following supporting information can be downloaded at: https://www.mdpi.com/article/10.3390/nu17081293/s1, Figure S1: Study flowchart; Figure S2: Generalized additive modelling of total calorie intake, parenteral calorie intake ratio (parenteral calorie intake/enteral calorie intake), insulin and phosphate requirements by group over time; Table S1: Daily medians and interquartile ranges of protein intake, calorie intake, calorie deficit and urea generation rate by group; Table S2: Daily medians and interquartile ranges of serum creatinine, creatinine clearance, serum urea and serum urea-to-creatinine ratio by group.

## Abbreviations

EE, Energy expenditure. ICU, intensive care unit. MODS, Multiple organ dysfunction syndrome. OR, odds ratio. SAPS III, Simplified Acute Physiology Score III. UCR, urea-to-creatinine ratio.
