# Supplementary material for: The Impact of Protein Feed on the Urea-to-Creatinine Ratio—A Retrospective Single-Center Study
Source: Nutrients. 2025 Apr 8;17(8):1293. doi: 10.3390/nu17081293 (PMC12029703; doi:10.3390/nu17081293)
Supplement: Supplementary file 1 [file nutrients-17-01293-s001.zip › nutrients-3549297-supplementary.pdf]

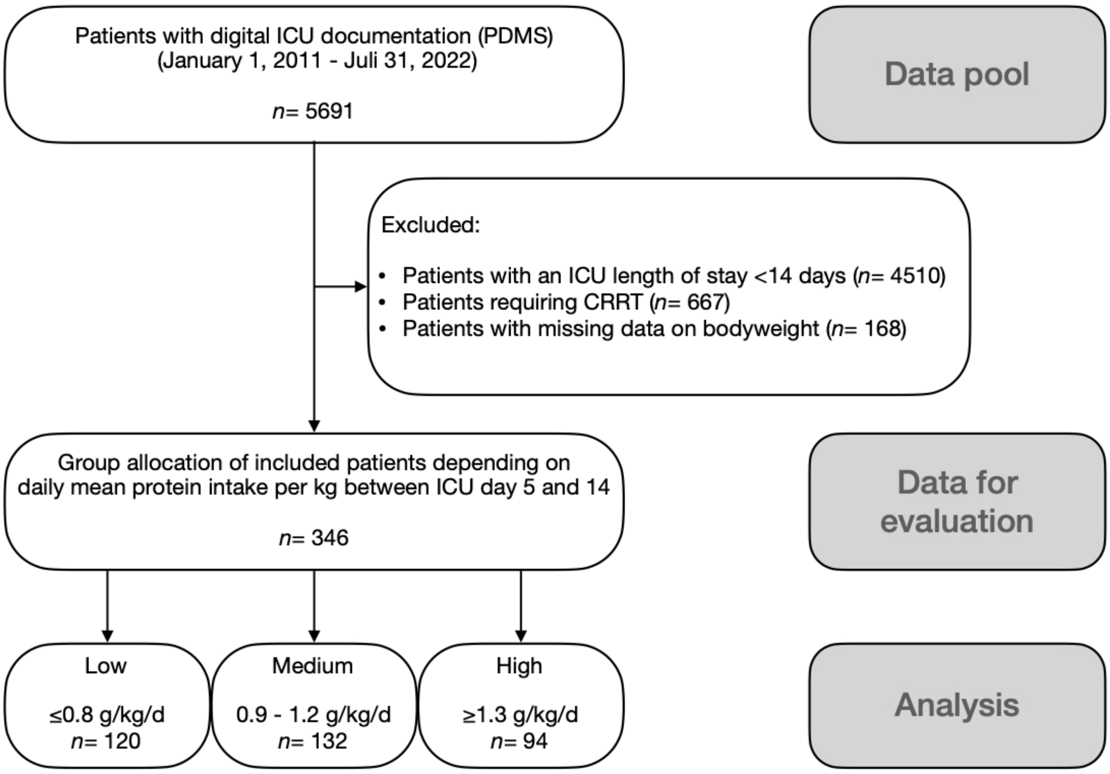

Supplemental Figure S1. Study flowchart.

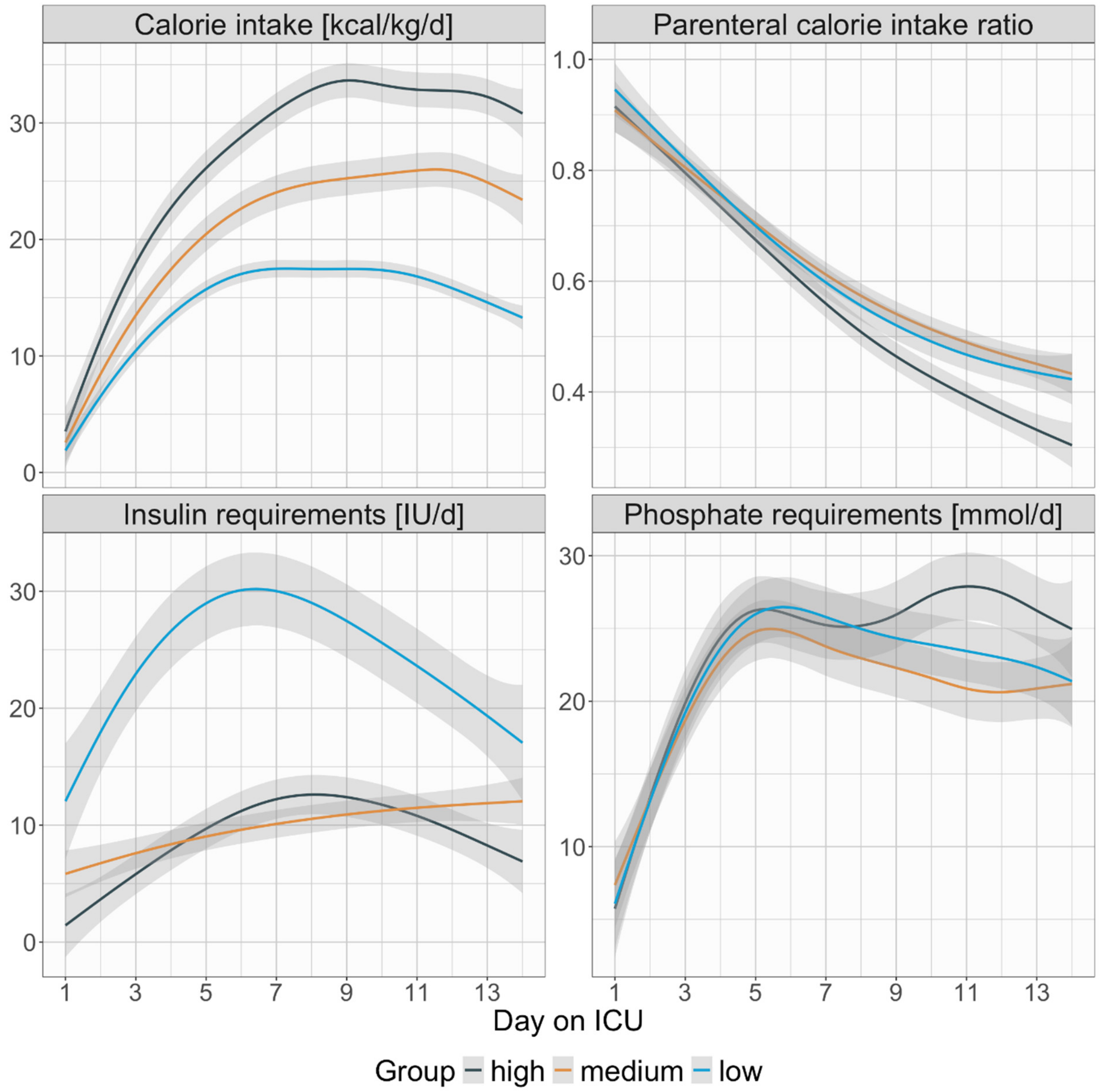

Supplemental Figure S2. Generalized additive modelling of total calorie intake, parenteral calorie intake ratio (parenteral calorie intake/enteral calorie intake), insulin and phosphate requirements by group over time. Grey shaded areas represent 95% confidence intervals.

Supplemental Table S1. Daily medians and interquartile ranges of protein intake, calorie intake, calorie deficit and urea generation rate by group.

	**Low**			**Medium**			**High**			
**Day**	**Median**	**Q1**	**Q3**	**Median**	**Q1**	**Q3**	**Median**	**Q1**	**Q3**	**p**
**Protein intake [g/kg]**										
1	0	0	0	0	0	0.02	0	0	0	0.996

2	0.33	0	0.61	0.52	0.2	0.74	0.7	0.28	0.99	<0.001
3	0.59	0.39	0.77	0.87	0.65	1.1	1.02	0.76	1.36	<0.001
4	0.69	0.59	0.88	0.94	0.75	1.11	1.19	1.02	1.55	<0.001
5	0.8	0.64	0.95	1.02	0.83	1.2	1.36	1.03	1.65	<0.001
6	0.8	0.64	0.96	1.1	0.89	1.26	1.46	1.28	1.77	<0.001
7	0.78	0.61	0.95	1.08	0.92	1.25	1.61	1.3	1.91	<0.001
8	0.76	0.59	0.9	1.09	0.91	1.32	1.71	1.36	2.01	<0.001
9	0.75	0.57	0.91	1.1	0.93	1.24	1.63	1.33	1.91	<0.001
10	0.77	0.59	0.92	1.1	0.96	1.24	1.59	1.34	1.97	<0.001
11	0.72	0.51	0.9	1.08	0.94	1.22	1.62	1.35	1.94	<0.001
12	0.69	0.43	0.87	1.08	0.89	1.22	1.63	1.31	1.9	<0.001
13	0.66	0.4	0.83	1.08	0.86	1.24	1.57	1.34	2.03	<0.001
14	0.55	0.33	0.77	1.04	0.8	1.23	1.42	1.18	1.74	<0.001
**Calorie intake [kcal/kg]**										
1	0.23	0	1.43	0.19	0	1.49	0.42	0	2.08	0.700
2	6.3	1.74	10.16	9.01	4.47	12.56	11.94	5.85	15.65	<0.001
3	9.81	6.98	13.39	13.31	10.52	16.81	16.84	12.21	23.47	<0.001
4	12.88	10.25	16.57	16	13.22	20.7	20.71	16.34	27.66	<0.001
5	15.51	11.93	20.23	19.86	16.14	24.16	22.51	18.18	32.72	<0.001
6	17.09	13.67	21.26	22.3	18.16	27.74	26.48	22.19	33.53	<0.001
7	17.76	13.72	21.74	23.44	19.01	27.92	29.36	25.34	35.37	<0.001
8	17.29	13.22	21.61	24.06	20.2	29.42	33	25.87	38.12	<0.001
9	17.84	13.02	21.32	24.15	20.74	29.05	31.07	25.94	38.71	<0.001
10	18.96	13.44	21.91	25.13	21.55	28.57	31.63	24.74	38.9	<0.001
11	17.3	12.74	21.58	24.26	20.87	28.03	33.04	24.87	37.48	<0.001
12	15.88	10.87	21.34	25.31	20.15	28.46	32.82	25.72	36.35	<0.001
13	15.4	10.39	20.03	24.88	20.19	29.28	32.94	27.45	37.33	<0.001
14	13.85	8.57	19.18	23.17	18.9	28.54	29.88	22.68	35.02	<0.001
**Calorie deficit [kcal]**										
1	1717.2	1325.7	2042.9	1612.1	1282.5	1845.4	1367.9	990.67	1609.9	<0.001
2	1337.7	814.13	1803.2	1087	639.37	1546.5	813.98	508.52	1228.8	<0.001
3	1035	513.72	1541.7	773.68	375.24	1140.8	473.92	116.24	796.32	<0.001
4	806.43	370.23	1284.2	638.55	249.07	979.48	255	50.23	547.54	<0.001

5	609.24	239.53	1072.3	479.97	56.93	811.84	172.16	-102.03	537.89	<0.001
6	523.29	237.32	1005	380.9	72.61	794.16	171.42	-158.49	480.49	<0.001
7	539.35	123.7	911.94	331.8	-12.05	850.43	62.47	-267.63	396.14	<0.001
8	591.46	296.67	1029	353.37	28.04	664.93	-92.38	-319.26	208.36	<0.001
9	518.69	237.84	1058.2	350.11	-5.48	626.05	35.21	-323.41	374.8	<0.001
10	550.49	109.78	909.51	281.38	11.72	573.01	-5.53	-377.32	442.08	<0.001
11	523.15	120.69	949.49	268.26	16.38	563.99	-10.73	-342.62	285.52	<0.001
12	581.61	152.76	915.29	280.53	-89.32	625.66	-5.75	-320.58	367.94	<0.001
13	610.79	213.68	940.62	229.96	-115.86	554.83	25.34	-336.96	372.68	<0.001
14	524.27	154.91	769.35	271.64	-115.07	715.43	87.87	-198.54	495.76	0.002
**Urea generation rate [g/d/70kg]**										
1	9.14	6.32	14.94	11.12	7.05	18.69	14.02	9.36	17.74	0.257
2	22.66	18.1	27.96	22.89	18.36	28.04	24.59	18.24	29.68	0.191
3	24.03	19.38	30.32	28.47	22.18	33.84	28.9	22.04	38.31	<0.001
4	25.59	19.83	34.36	30.73	24.19	37.99	34.57	27.07	44.83	<0.001
5	29.5	21.99	37.07	33.94	27.08	42.49	39.44	31.44	45.43	<0.001
6	31.13	23.36	39.85	36.94	27.8	46.19	43.09	34.58	52.34	<0.001
7	30.81	23.59	38.94	38.02	29.37	46.14	46.1	36.55	56.4	<0.001
8	32.49	22.4	40.96	40.59	29.75	49.32	48.74	38.34	58.64	<0.001
9	30.01	21.4	40.6	39.97	30.77	47.82	45.89	38.77	56.56	<0.001
10	28.68	20.15	39.24	39.02	30.85	45.63	46.79	39.36	55.12	<0.001
11	28.3	22.18	38.45	40.11	30.98	46.73	45.62	36.79	56.9	<0.001
12	27.69	20.88	37.1	37.49	29.49	45.81	44.52	38.25	53.44	<0.001
13	27	19.71	33.33	36.27	27.47	44.29	47.01	39.27	54.71	<0.001
14	24.93	18.51	34.88	35.01	26.43	43.18	43.63	36.61	53.84	<0.001

Supplemental Table S2. Daily medians and interquartile ranges of serum creatinine, creatinine clearance, serum urea and serum urea-to-creatinine ratio by group.

	**Low**			**Medium**			**High**			
**Day**	**Median**	**Q1**	**Q3**	**Median**	**Q1**	**Q3**	**Median**	**Q1**	**Q3**	**p**
**Creatinine [mg/dl]**										
1	0.98	0.84	1.33	0.94	0.81	1.17	0.88	0.71	1.11	0.043

2	1.05	0.86	1.45	1.04	0.87	1.27	0.99	0.74	1.22	0.022
3	1.12	0.91	1.6	1.03	0.82	1.32	0.96	0.72	1.25	0.002
4	1.12	0.79	1.53	1.02	0.76	1.35	0.9	0.68	1.24	0.005
5	1.02	0.8	1.4	0.94	0.72	1.29	0.86	0.63	1.18	0.009
6	1.03	0.8	1.35	0.86	0.65	1.27	0.84	0.61	1.15	0.002
7	0.99	0.73	1.3	0.86	0.66	1.19	0.79	0.56	1.06	0.001
8	0.9	0.71	1.24	0.84	0.64	1.16	0.74	0.54	0.99	0.001
9	0.86	0.7	1.18	0.84	0.61	1.15	0.72	0.51	0.98	0.002
10	0.83	0.69	1.12	0.82	0.62	1.16	0.71	0.51	0.87	0.001
11	0.82	0.65	1.09	0.8	0.6	1.11	0.7	0.53	0.85	0.004
12	0.81	0.64	1.06	0.76	0.58	1.04	0.69	0.51	0.89	0.003
13	0.79	0.66	1.06	0.73	0.59	0.98	0.66	0.51	0.86	0.001
14	0.75	0.64	0.99	0.71	0.57	0.98	0.66	0.48	0.8	0.002
**Creatinine Clerarance [ml/min/1.73m^2]**										
1	87.23	46.06	114.6	73.53	61.73	111.54	77.58	51.23	87.42	0.952
2	61	39.3	119.06	73.37	34.94	106.65	50.61	28.54	75.73	0.033
3	77.42	54.31	125.34	86.78	63.54	129.16	78.95	54.35	116.79	0.459
4	85.77	54.59	136.19	90.21	61.15	136.84	92.49	58.94	122.4	0.618
5	89.51	55.82	132.69	103.4	62.85	141.47	102.39	61.6	135.43	0.380
6	94.74	56.21	127.33	102.97	66.85	147.32	103.15	66.58	135.56	0.453
7	90.52	62.67	136.16	103.04	65.92	147.98	104.52	65.67	136.55	0.421
8	92.8	66.56	136.42	107.49	65.64	160.78	110.46	71.98	146.96	0.193
9	101.09	68.83	133.55	106.24	74.35	156.85	111.25	75.55	148.66	0.255
10	106.67	70.04	143.3	111.9	66.43	155.14	107.88	75.07	143.59	0.711
11	101.63	68.94	142.9	108.03	68.78	160.48	112.34	76.69	147.24	0.721
12	105.16	75.57	141.93	114.07	74.78	162.77	108.48	80	143.06	0.594
13	109.11	72.9	149.53	116.01	75.63	158.59	114.9	82.13	150.69	0.688
14	105.26	71.28	146.33	117.43	79.65	170.44	120.13	79.23	149.7	0.420
**Urea [mg/dl]**										
1	39.25	30.97	58.38	39.7	27.38	48.43	36.75	27.9	53.45	0.555
2	44	29.9	59.5	41.9	30.8	56.35	43.3	30.3	61.05	0.960
3	49.25	34.72	70.8	45.5	35.3	68.7	50.2	34.4	72.9	0.734
4	53.9	36.6	85.5	53	38.8	78.55	50.8	37.2	82.9	0.892

5	59.2	39.95	91.35	56.45	41.08	80.25	51.95	39.4	90.3	0.594
6	60.5	42.25	88.15	57.8	41.73	80.72	54.85	44.5	90.62	0.755
7	60	41.6	91.85	55.8	39.73	85.55	60.2	44	85.3	0.494
8	59.25	40.12	87.28	53.15	40.53	87.95	61.15	46.42	86.21	0.333
9	57.3	39.5	82.7	55	40.45	93.3	62	43.5	88.1	0.313
10	54.4	40.15	77.1	54.7	38.35	92.22	60.8	45.2	85.2	0.327
11	52.95	38.67	76.08	51.9	37.5	88.1	57.7	44.1	85.3	0.123
12	51.35	34.95	73.6	52.8	37.7	81.5	56.05	46.28	80.47	0.125
13	49.5	36.25	66.2	51	38.08	78.05	55.45	43.28	78.62	0.064
14	44.2	31.9	63.1	48.25	34.58	71.5	57.7	44.58	76.5	0.001
**Urea-to-creatinine ratio**										
1	38.74	31.42	53.06	38.11	31.32	50.56	40.3	32.67	63.11	0.186
2	37.3	29.57	50.48	38.88	31.03	51.71	42.92	32.76	66.29	0.042
3	41.14	35.04	52.42	45.23	35.85	59.61	53.09	35.33	70.3	0.015
4	49.44	38.18	62.32	54.27	42.49	69.28	56.09	41.28	82.5	0.015
5	56.56	44.42	74.07	59.14	47.25	76.25	61.9	47.05	90.43	0.143
6	60.77	46.75	75.34	65.2	50.84	78.96	70.71	55.55	92.47	0.002
7	63.5	50.12	74.42	68.61	51.65	83.02	77.75	59.2	100.87	<0.001
8	61.45	49.7	75.16	66.87	53.44	84.66	84.65	62.46	108.87	<0.001
9	61.4	49.94	81.1	70.34	56.23	86.74	82.86	63.72	112.95	<0.001
10	63.88	49.26	81.37	69.81	54.27	86.45	84	63.66	110.83	<0.001
11	62.52	47.44	82.68	70.6	55.96	90.36	88.92	67.17	109.8	<0.001
12	61.79	47.16	81.67	69.14	56.27	87.71	89.48	68.27	110.05	<0.001
13	59.44	47.47	79.83	69.4	56.03	89.08	93.06	65.24	113.11	<0.001
14	59.63	43.74	76.56	69.28	56.33	86.74	93.76	75.97	119.95	<0.001
